# Ground truth clustering is not the optimum clustering

**DOI:** 10.1038/s41598-025-90865-9

**Published:** 2025-03-17

**Authors:** Lucia Absalom Bautista, Timotej Hrga, Janez Povh, Shudian Zhao

**Affiliations:** 1 University of Sevilla, C. San Fernando 4, Seville, 41004 Spain; 2 Present Address: Institut für Mathematik, Alpen-Adria-Universität Klagenfurt, Universitätstraße 65-67, Klagenfurt, 9020 Austria; 3 Faculty of Mechanical Engineering, University of Ljubljana, Aškerčeva 6, Ljubljana, 1000 Slovenia; 4 Rudolfovo – Science and technology center Novo Mesto, Podbreznik 15, Novo Mesto, 8000 Slovenia; 5 Department of Mathematics, KTH - Royal Institute of Technology, Lindtstedtsvägen 25, Stockholm, 100 44 Sweden

**Keywords:** Minimum sum-of-squares clustering, Ground truth clustering, Extrinsic measures, Intrinsic measures, Engineering, Mathematics and computing

## Abstract

Data clustering is a fundamental yet challenging task in data science. The minimum sum-of-squares clustering (MSSC) problem aims to partition data points into *k* clusters to minimize the sum of squared distances between the points and their cluster centers (centroids). Despite being NP-hard, solvers exist that can compute optimal solutions for small to medium-sized datasets. One such solver is SOS-SDP, a branch-and-bound algorithm based on semidefinite programming. We used it to obtain optimal MSSC solutions (optimum clusterings) for various *k* across multiple datasets with known ground truth clusterings. We evaluated the alignment between the optimum and ground truth clusterings using six extrinsic measures and assessed their quality using three intrinsic measures. The results reveal that the optimum clusterings often differ significantly from the ground truth clusterings. Additionally, the optimum clusterings frequently outperform the ground truth clusterings, according to the intrinsic measures that we used. However, when ground truth clusters are well-separated convex shapes, such as ellipsoids, the optimum and ground truth clusterings closely align.

## Introduction

### Motivation

Data science is strongly related to the progress in multiple sub-fields of mathematics and computer science. Understanding the data and finding hidden relations between data instances or variables are getting more and more important, especially in the era of Big Data and Artificial Intelligence. Grouping the data instances according to their inner similarity is called clustering analysis^1^, Chapter 10.3^2,3^. A vast amount of literature is devoted to this problem, covering several different aspects. Nevertheless, two important questions always need to be answered: (i) what is the number of groups that we want to cluster the data into? (ii) what is the similarity measure that will be used to compute the similarity of two data points and consequently to define groups of similar data, i.e., the clusters? Usually, these two questions are strongly related, i.e., the number of clusters and the clustering are strongly dependent on the underlying similarity measure.

In some cases, we know in advance the actual number of groups into which the data should be clustered, e.g., based on some domain-specific knowledge. In these cases, the main task is to compute an assignment of the data points into this number of groups, which is also called the *k*-clustering problem. However, this is a rather rare situation. Usually, the number of clusters has to be determined during the clustering analysis. Some methods, such as the hierarchical clustering methods, determine the number of clusters during the clustering process. Other methods, such as the *k*-means or the *k*-median clustering algorithms, require this number as input, and there are many different criteria and techniques that can be used to determine the appropriate number of clusters, e.g., the cross-validation, the “Elbow” method, the gap statistic, the silhouette method, etc., see^4,5^.

Clustering is often part of exploratory research and, in general, there is not only one good/optimum clustering. The selection of similarity measures, number of groups, and good assignment into clusters are often done iteratively, based on an increasing understanding of the data and partial clustering results. Many algorithms for clustering analysis are known in the literature. Usually, they iteratively define groups of similar data points by following some principles that should hold for good clustering, e.g., cluster homogeneity, cluster completeness, possessing a rag bag cluster, *n*-invariance, see e.g.,^6^. In each iteration, they improve the clustering such that the selected criteria are improved. Once there is no more improvement or the maximum number of iterations is reached, the algorithm terminates.

In a very complex situation where there are many criteria and principles for good clustering, and consequently many different algorithms for computing such solutions, usually leading to many different clustering solutions for the same dataset, the question naturally arises as to what is a ground truth, i.e., which groups are most natural, which data points really belong to the same group. Very often such clustering is not known, but for some datasets it is provided by the data provider. If we know the ground truth clustering, we can compare the clustering computed by different algorithms with it. This was the main motivation for us. We focused on an exact mathematical programming formulation of the clustering problem, solved it with available global optimization algorithms, and compared the optimum solution with the ground truth clustering.

### The data clustering problem

The data clustering problem can be formulated as follows: given a set of data points ${\mathcal {P}}=\{p_1,p_2,\ldots ,p_n\}\subset \mathbb {R}^m$, the objective is to find the cluster number *k* and an assignment $\chi :{\mathcal {P}}\rightarrow \{1,2,\ldots ,k\}$ such that $\chi (p_i)=\chi (p_j)$ for any pair of (very) data similar points $p_i$ and $p_j$ ($p_i \ne p_j$). For any *k* assignment $\chi$ we can define the *i*-th cluster, implied by $\chi$ as $C_i=\chi ^{-1}(i)$ and the corresponding clustering is ${\mathcal {C}}=\{C_1,\dots , C_k \}$.

Even if we already know the number *k*, there are still many possibilities for how to measure (high) similarity between the data points and how to compute good clustering ${\mathcal {C}}$. In this paper, we will focus on Euclidean distance measure between the two data points. The closer the points are in the Euclidean distance, the more similar they are. We evaluate each clustering by considering the total sum of squares of distances between the data points and the centroid of the corresponding cluster. This means that: (i) for each cluster we compute its centroid (the vector sum of all data points from this cluster, divided by the cardinality of this cluster). (ii) for each data point we compute the square Euclidean distance between it and the centroid of the cluster to which this data point is assigned. The sum of squares of these distances across all data points is a measure of the quality of the clustering. The smaller it is, the better the clustering.

We can formulate the problem of minimizing the sum of squares of distances as a mathematical optimization problem, where the decision variables represent assignments $\chi$ and the objective function is the sum of squares of distances. This yields the so-called minimum sum-of-squares clustering (MSSC) problem, formally introduced in “Mathematical programming formulation for MSSCproblem” section. This problem is well-known in the literature^7,8^ as it arises in a wide range of applications, for example, image segmentation^9,10^, biology^11^ and document clustering^12^. It is an NP-hard problem^13,14^, which means that there is no polynomial time algorithm to solve it to optimality (unless P=NP). Nevertheless, we can still solve it for small datasets, e.g., by using the exact solver SOS-SDP^15^. The announcement of this solver was actually the trigger for our research. We decided to solve the MSSC problem to optimality for a number of small datasets for which the ground truth clustering ${\mathcal {C}}_{true}$ and the true number of clusters $k_{true}$ were known - usually provided by the dataset provider.

Clustering performance can be quantified by a number of metrics. There are mainly two types of metrics to evaluate this performance. To compare different clustering methods, we use intrinsic and extrinsic measures. Extrinsic measures require ground truth labels, while intrinsic measures do not.

The datasets with a ground truth clustering are actually the classification datasets, where the classification labels are considered as the ground truth label. This idea was elaborated in^16^, where the authors show that this approach requires careful attention since the class labels are assigned based on the properties of each individual data point, while the clusterings take into account relations between the data. Our paper is aligned with this observation and provides a deeper understanding of this issue.

A ground truth-based comparative study was done in^17^, where the authors did not solve an exact clustering problem (like MSSC) but rather compared five widely known approximate algorithms for data clustering on seven published micro-array gene expression datasets and one artificial dataset. The performances of these algorithms were assessed with several quantitative performance measures, which are different from the measures that we use in this paper.

In^18^ the authors introduced a population-based metaheuristic algorithm to solve MSSC approximately, which performs like a multi-start *k*-means. They demonstrate that this algorithm outperforms all recent state-of-the-art approximate algorithms for MSSC in terms of local minima and the computed clusters are closer to the ground truth compared to the clusters computed by other algorithms, on the artificial Gaussian-mixture datasets.

In^6,16,17^, the ground truth labels and the extrinsic metrics were used to validate the quality of clusterings and compare different clustering methods. The ground truth labels have shown limitations in many instances for detecting the hidden pattern of the sample and they can produce misleading information when used as true ground labels for measuring the quality of clusterings^16^.

The clustering problem and its variants remain subject of intensive research. The multi-view clustering problem recently received lot of research attention. It can be formulated as a problem of non-negative matrix factorization of several view matrices with joint consensus matrix^19–21^. This optimization problem is again very hard, so the authors devised their own algorithms, for which they proved a convergence, but not necessarily to a global optimum. The resulting local optima were used to extract multi-view clusterings, and these clusterings were compared with the ground truth labelings using some of the well-known similarity measures, like accuracy and normalized mutual information (NMI). A similar approach was used for complex data clustering^22^, graph-based subspace clustering^23^, and representation learning^24^. In this work, the authors do not use the clusterings based on the global optimum of mathematical programming formulations of the basic problems, as we do in this paper, since they do not compute the global solutions. Actually, they made no attempt to compute the global optima of the mathematical programming formulation of the clustering problem, as we do, probably because these problems are very hard to solve globally.

### Our contributions

The main goal of this paper is to solve exactly the MSSC problem on a large list of small or medium size datasets available in the literature for which the true number of clusters $k_{true}$ and the ground truth clustering ${\mathcal {C}}_{true}$ are known, and to check how the MSSC optimum clustering ${\mathcal {C}}_{MSSC}$, i.e. the clustering that corresponds to an optimum solution of MSSC aligns with the ground truth clustering. More precisely, we

- solve the mathematical programming formulation of clustering problem MSSC to optimality for values of *k* with $|k-k_{true}|\le 2$ for 12 real datasets and for 12 artificial datasets, obtained from https://github.com/deric/clustering-benchmark/tree/master/src/main/resources/datasets, which are small enough and for which the ground truth clustering ${\mathcal {C}}_{true}$ is available. This data is available under the open data standards, together with the optimum solutions, see^25^;
- compare the MSSC optimum clusterings ${\mathcal {C}}_{MSSC}$ with the ground truth clusterings ${\mathcal {C}}_{true}$ by computing a number of extrinsic measures: Adjusted Mutual Information (AMI), Adjusted Random Score (ARS), Homogeneity (h), Completeness (c), normalized mutual information (NMI) and Fowlkes-Mallows scores (FMS);
- additionally evaluate the quality of the optimum MSSC clusterings ${\mathcal {C}}_{MSSC}$ and of the ground truth clusterings ${\mathcal {C}}_{true}$ by computing three intrinsic measures: the Calinski-Harabasz Criterion (CHC), the Davies Bouldin Index (DBI), and the Silhouette Evaluation Score $S_{score}$;
- provide a better understanding of why and when the optimum clustering aligns with the ground truth clustering.

Our analysis shows that

- the optimum clustering (i.e., the optimum solution of MSSC) can be computed if the number of data points times the number of clusters *k* is approximately below 1000;
- the optimum clusterings ${\mathcal {C}}_{MSSC}$ usually significantly differ from the ground truth clustering in the following manner: the values of the extrinsic measures are often far below the optimum, which is 1 and would be achieved if the ground truth and the optimum clustering would be the same. Additionally, the values of extrinsic measures, evaluated at ${\mathcal {C}}_{MSSC}$ and corresponding to $k_{true}$ are rarely optimum, i.e., other *k* often gives better values of these measures;
- the ground truth clusterings are usually much worse compared to the optimum clusterings by considering the three intrinsic quality measures;
- when the ground truth clustering has natural expected geometry, i.e., the clusters have the form of convex sets, e.g., ellipsoids, which are well separated from each other, then the ground truth clustering is very similar to the optimum clustering.

These observations are not unexpected but, to the best of our knowledge, have never been shown so explicitly.

The main innovation of this paper therefore lies in computing the global optimum of the MSSC formulation for the clustering problem and rigorously comparing it to the ground truth clustering provided by domain specialists who supplied the original data. Through a detailed quantitative analysis, we demonstrate that these two clusterings exhibit significant discrepancies, providing robust evidence that, in general, they are far from being similar.

### Outline

The rest of the paper is structured as follows. In “Mathematical programming formulation for MSSCproblem” section, we present the SDP relaxation for MSSC problem and explains hows the exact solver SOS-SDP works. “Quality measures” section introduces all the metrics to compare the quality of different clustering and the metrics of all clustering results are presented in “Numerical results” section. “Discussion” section analyzes the numerical results and “Conclusions” section concludes the paper and discusses future work.

### Notation

The Euclidean distance between two points $p,q\in \mathbb {R}^m$ is denoted by $d_E$ and defined as follows $d_E(p,q)=\Vert p-q\Vert =\sqrt{\sum _{i}^m (p_i-q_i)^2}$. We use [*n*] to denote the set of integers $\{1,\dots ,n\}$. The trace of matrix *X* is denoted by $\text {tr}(X)$. The space of symmetric matrices is equipped with the trace inner product, which for any $X, Y \in {{\mathcal {S}}}^{n}$ is defined as ${\langle X,Y \rangle }:= \text {tr}(XY)$. The associated norm is the Frobenius norm $\Vert X\Vert _F := \sqrt{\text {tr}(X^2)}=\sqrt{\sum _{ij} x_{ij}^2}$. The cone of symmetric positive semidefinite matrices of order *n* is denoted by ${{\mathcal {S}}}_+^n :=\{X \in {{\mathcal {S}}}^n\mid X\succeq {\textbf{0}} \}$.

We denote by ${\textbf{e}}_n$ the vector of all ones of length *n*. In case that the dimension of ${{\textbf{e}}}_n$ is clear from the context, we omit the subscript. The operator $\text {diag}:\mathbb {R}^{n\times n} \rightarrow \mathbb {R}^n$ maps a square matrix to a vector consisting of its diagonal elements. Its adjoint operator is denoted by $\text {Diag}:\mathbb {R}^n \rightarrow \mathbb {R}^{n\times n}$. The rank of matrix *X* is denoted by $\text {rank}(X)$. The average value of components of vector *v* is denoted by $\text {avg}(v)$.

## Mathematical programming formulation for MSSC problem

In this paper, we use the formulation of the MSSC problem as a mathematical optimization problem in binary variables, which are subject to linear constraints, and with a non-convex objective function that represents the sum of squares of Euclidean distances between the data points and the centroids of the clusters to which the points correspond.

For given set of data points ${\mathcal {P}}=\{p_1,p_2,\ldots ,p_n\}\subset \mathbb {R}^m$ and given integer *k* we define *k*-clustering as an assignment $\chi :{\mathcal {P}}\rightarrow \{1,2,\ldots ,k\}$. The data points that are mapped to integer $1\le i\le k$ are called the *i*-th cluster. The assignment is nontrivial if there is no empty cluster. We can represent each nontrivial assignment $\chi$ by a matrix $X\in \{0,1\}^{n\times k}$ such that $x_{ij}=1$ if and only if $\chi (p_i)=j$. Therefore, the row-sums of *X* must be equal to 1 (each data point is assigned to exactly one cluster) and the column-sums of *X* must be at least one (otherwise the assignment is not nontrivial). Therefore, the minimum sum-of-squares clustering (MSSC) problem for a fixed *k* can be formulated as a non-linear integer programming problem (Vinod^8^, Rao^7^):

MSSC $$\begin{aligned} \begin{aligned} \min ~&\sum ^{n}_{i=1}\sum ^k_{j=1}x_{ij}\Vert p_i - c_{j}\Vert ^2\\ \text {s.t.}~~&\sum ^k_{j=1}x_{ij} = 1, \forall i \in [n],\\&\sum ^n_{i=1}x_{ij} \ge 1, \forall j \in [k],\\&x_{ij} \in \{0, 1\}, \forall i \in [n] ~\forall j \in [k], \\&c_j \in \mathbb {R}^m, \forall j \in [k], \end{aligned} \end{aligned}$$

where $c_j$ is the centroid of the *j*-th cluster and can be substituted as

1 $$\begin{aligned} c_j = \frac{\sum \limits ^n_{i=1} x_{ij} p_i}{\sum \limits ^n_{i=1} x_{ij}}, \quad \forall j \in [k]. \end{aligned}$$

This formula can be derived by setting the gradient with respect to the variable $c_j$ of the objective function to zero. We obtain

$$\begin{aligned} \sum \limits ^n_{i=1} x_{ij}(-2p_i + 2c_j) = 0. \end{aligned}$$

By expressing the varibble $c_j$ we get the formula (1).

Peng et al.^26^ introduced equivalent formulations for (MSSC) by introducing substitution $Z:=X(X^\top X)^{-1}X^\top$:

2 $$\begin{aligned} \begin{aligned} \min ~&-\langle W, Z \rangle \\ \text {s.t.}~~&Ze = e, \\&\text {tr}(Z) = k,\\&Z \succeq 0,~Z\ge 0,\\&\textrm{rank}(Z)=k, \end{aligned} \end{aligned}$$

where $W = (W_{ij}) \in {{\mathcal {S}}}^n$ with $W_{ij} = p_i^\top p_j$. If we know an optimum matrix *Z* for (2), we can get back the optimum clustering matrix *X* for (MSSC) by using the fact that $z_{ij}>0$ if and only if the data vertices $p_i$ and $p_j$ are in the same cluster. So we can put $p_1$ to cluster 1 and the same we do with all $p_i$ with $z_{1i}>0$. The first data point that remains unassigned is then put to cluster 2 and the same all data points with $z_{2i}>0$. This is repeated until all data points are assigned.

MSSC in formulation (2) remains NP-hard. However, this formulation opens new possibilities to solve (MSSC) to optimality. Piccialli et al^15^ introduced an SDP relaxation for (2) by eliminating the constraint $\textrm{rank}(Z)=k$, which can be solved to arbitrary precision with state-of-the-art SDP solvers (e.g.,^27,28^). The SDP relaxation serves as a model to generate lower bounds in the exact methods such as the branch-and-bound algorithm. The SOS-SDP algorithm from^15^ is an implementation of such branch-and-bound algorithm based on this relaxation, which was additionally significantly strengthened with the following cutting planes:

- *Triangle inequalities*, which are based on the observation that if the points *i* and *j* are in the same cluster and the points *j* and *h* are in the same cluster, then the points *i* and *h* must necessarily belong to the same cluster:
  $$\begin{aligned} Z_{ij} + Z_{ih} \le Z_{ii} + Z_{jh}, ~\forall i, j, h \in [n],~ i\ne j\ne h. \end{aligned}$$
- *Pair inequalities*
  $$\begin{aligned} Z_{ij} \le Z_{ii},~Z_{ij} \le Z_{jj},~\forall i, j \in [n],~i \ne j \end{aligned}$$
  that every feasible solution of (2) satisfies.
- *Clique inequalities*
  $$\begin{aligned} \sum _{(i,j)\in I\times I,i<j} Z_{ij} \ge \frac{1}{n-k+1},~\forall I \subset [n],~|I| =k+1, \end{aligned}$$
  enforcing that for any subset *I* of $k + 1$ points at least two points have to be in the same cluster.

The branch-and-bound algorithm also demands strong upper bounds for the optimum value, which are usually obtained by computing good feasible solutions, in our case good assignments to clusters. The SOS-SDP algorithm computes these bounds by using the COP *k*-means algorithm from^29^, which is a special variant of the well-known *k*-means heuristics.

## Quality measures

Let denote by ${\mathcal {C}}_{true}:=\{C_{true,1},\dots ,C_{true,k} \}$ the clustering with cluster number *k*, given by the data provider - we call it the ground truth clustering, and ${\mathcal {C}}_{MSSC}=\{C_{MSSC,1},\dots ,C_{MSSC,k} \}$ the exact clustering, i.e., the optimum solution of (MSSC), computed in our case by the SOS-SDP algorithm.

To assess the quality of the computed clusterings, we use both external (extrinsic) and internal (intrinsic) measures).

Using the former, we will compare the computed clusters ${\mathcal {C}}_{MSSC}$ with the ground truth clusters ${\mathcal {C}}_{true}$, while the latter do not require ground truth clustering because for the computed clusters they measure certain criteria such as cluster compactness. There is a long list of possible measures, see for example^6^, but we restrict ourselves only to those implemented in the Python library scikit-learn (https://scikit-learn.org/stable/modules/clustering.html#clustering-performance-evaluation), which we chose for our computations, see also^30^. We include the definition for each method and original reference (see Table 1) for each method in this section, but more details can be also found from textbooks such as^31,32^.

**Table 1:** Summarized information of extrinsic (upper part) and intrinsic (lower part) measures.

Name	Abbreviation	Reference
Adjusted mutual information	AMI	^33^
Adjusted random score	ARS	^34,35^
Homogeneity	*h*	^36^
Completeness	*c*	^36^
V-measure	*v*	^36^
Fowlkes-Mallows scores	FMS	^37^
Calinski-Harabasz criterion	CHC	^38^
Davies Bouldin Index	DBI	^39^
Silhouette evaluation score	$S_{score}$	^40^

### Extrinsic measures

The extrinsic methods in general evaluate how two different clusterings match. In our case, we use them to assess how the clustering computed by solving the MSSC to optimum (we call this clustering the optimum clustering and denote it by ${\mathcal {C}}_{MSSC}$) matches with the ground truth clustering, i.e., the clustering provided by the data provider algorithm (we denote it by ${\mathcal {C}}_{true}$). We use six measures from the Python scikit-learn package^30^. For the sake of completeness, we provide here a short description for each of them, based on the well-known literature^34,36,37,39–42^.

- *Mutual Information*: The mutual information^41,42^ is also known as the information gain and is computed by
  3 $$\begin{aligned} \begin{aligned}&\textrm{ MI}({\mathcal {C}}_{true},{\mathcal {C}}_{MSSC})\\&\quad =\sum _{C_{true,i} \in {\mathcal {C}}_{true}} \sum _{C_{MSSC,j} \in {\mathcal {C}}_{MSSC}} \frac{|C_{true,i}\cap C_{MSSC,j}|}{(\sum _{C_{true,i} \in {\mathcal {C}}_{true}} |C_{true,i}|)^2 } \log \frac{|C_{true,i} \cap C_{MSSC,j}|}{|C_{true,i}||C_{MSSC,j}|}, \end{aligned} \end{aligned}$$
  where $k_{true}$ and $k_{MSSC}$ are the number of clusters in ${\mathcal {C}}_{true}$ and ${\mathcal {C}}_{MSSC}$, respectively, and $C_{\cdot ,i}$ is the *i*-th cluster in the clustering assignment ${\mathcal {C}}_{\cdot }$. The score is nonnegative, and a higher value indicates a higher similarity between the two clusterings.
- *Adjusted Mutual Information (AMI)*:
  The adjusted mutual information score for ${\mathcal {C}}_{true}$ and ${\mathcal {C}}_{MSSC}$ is computed as^33^
  $$\begin{aligned} \textrm{AMI}({\mathcal {C}}_{true}, {\mathcal {C}}_{MSSC}) = \frac{\textrm{MI}({\mathcal {C}}_{true}, {\mathcal {C}}_{MSSC}) - \mathbb {E}[\textrm{ MI}]}{ \text {avg}(H({\mathcal {C}}_{true}), H({\mathcal {C}}_{MSSC})) - \mathbb {E}[\textrm{ MI}]}, \end{aligned}$$
  where $\text {avg}(\cdot )$ denotes the arithmetic average and $H({\mathcal {C}})$ is the entropy of a clustering ${\mathcal {C}}$:
  4 $$\begin{aligned} H({\mathcal {C}}) = \sum _{C_i \in {\mathcal {C}}} -\frac{|C_i|}{\sum _{C_j \in {\mathcal {C}}} |C_j|} \log \frac{|C_i|}{\sum _{C_j \in {\mathcal {C}}} |C_j|}, \end{aligned}$$
  and $\mathbb {E}(\textrm{ MI})$ is the expected mutual information between two random clusterings.
  The AMI score is between $-1$ and 1. It is 1 when the compared clusterings are identical.
- *Adjusted Random Score (ARS)*: ARS^34,35^ computes the proportion of pairs of data points that are in the same cluster or in a different cluster in both clusterings (in our case: ${\mathcal {C}}_{true}$ and ${\mathcal {C}}_{MSSC}$) and normalize this proportion by using the expected similarity specified by a random model, usually based on the generalized hypergeometric distribution. It is computed as
  $$\begin{aligned} \textrm{ARS} = \frac{\textrm{ RI} - \mathbb {E}[\textrm{ RI}]}{\max (\textrm{ RI}) - \mathbb {E}[\textrm{ RI}]}, \end{aligned}$$
  where random index $$\textrm{ RI}= \frac{a+b}{\left( {\begin{array}{c}n\\ 2\end{array}}\right) }$$ and *a* and *b* are the numbers of pairs of elements that are in the same cluster in ${\mathcal {C}}_{true}$ and in the same cluster in ${\mathcal {C}}_{MSSC}$ and the number of pairs of elements that are in different clusters in ${\mathcal {C}}_{true}$ and in different clusters in ${\mathcal {C}}_{MSSC}$, respectively. The value $\mathbb {E}[\textrm{ RI}]$ is the expected value of $\textrm{ RI}$ between two random clusterings. The score is between $-1$ and 1. It is 1 when the compared clusterings are identical.
- *Homogeneity* (*h*), *Completeness* (*c*) and *V-measure* (*v*)^36^:
  Homogeneity measures how homogeneous the clusters are in ${\mathcal {C}}_{MSSC}$, i.e., whether the data points from the same cluster from ${\mathcal {C}}_{MSSC}$ (mostly) belong to the same cluster in ${\mathcal {C}}_{true}$.
  Completeness measures how many similar samples are put together by the clustering algorithm, i.e., if the data points belonging to the same cluster from ${\mathcal {C}}_{true}$ are also in the same cluster in ${\mathcal {C}}_{MSSC}$.
  The homogeneity *h* and completeness *c* are between 0 and 1 and are defined as
  $$\begin{aligned} h= 1- \frac{H({\mathcal {C}}_{true}|{\mathcal {C}}_{MSSC})}{H({\mathcal {C}}_{true})}, ~c= 1- \frac{H({\mathcal {C}}_{true}|{\mathcal {C}}_{MSSC})}{H({\mathcal {C}}_{MSSC})}, \end{aligned}$$
  where $H({\mathcal {C}}_1\mid {\mathcal {C}}_{2})$ is the conditional entropy of the clusters in ${\mathcal {C}}_1$ given the clustering prediction ${\mathcal {C}}_2$ (recall, *n* is the number of data points)
  5 $$\begin{aligned} H({\mathcal {C}}_{1} \mid {\mathcal {C}}_{2})= - \sum _{C_{2,j}\in {\mathcal {C}}_{2}} \sum _{C_{1,i} \in {\mathcal {C}}_{1}} \frac{|C_{2,j}\cap C_{1,i}|}{n} \log \frac{|C_{2,j}\cap C_{1,i}|}{\sum _{C_{1,s} \in {\mathcal {C}}_{1}} |C_{2,j}\cap C_{1,s}|}, \end{aligned}$$
  and *H* is entropy, defined above.
  The V-measure *v* is the weighted harmonic mean between the homogeneity and the completeness:
  6 $$\begin{aligned} v = \frac{(1 + \beta ) \cdot h \cdot c}{\beta \cdot h + c}, \end{aligned}$$
  where $\beta \ge 0$. When $\beta <1$, more weights are attributed to homogeneity; when $\beta >1$ more weights are attributed to completeness; when $\beta =1$, the score is also called *normalized mutual information (NMI)*, which we report in tables of “Numerical results” section:
  7 $$\begin{aligned} \textrm{NMI} = \frac{2 \cdot h \cdot c}{ h + c}, \end{aligned}$$
- *Fowlkes-Mallows scores (FMS)*^37^: The Fowlkes-Mallows score (FMS) is defined as the geometric mean between pairwise precision and recall, using True Positive (TP), False Positive (FP), and False Negative (FN):
  $$\begin{aligned} \textrm{FMS} = \frac{\textrm{TP}}{ \sqrt{(\mathrm {TP + FP}) \cdot (\mathrm {TP + FN})}}, \end{aligned}$$
  where TP is the number of pairs of points that belong to the same clusters in both ${\mathcal {C}}_{true}$ and ${\mathcal {C}}_{MSSC}$, FP is the number of pairs of points that belong to the same clusters in ${\mathcal {C}}_{true}$ and to different clusters in ${\mathcal {C}}_{MSSC}$, and FN is the number of pairs of points that belongs to the same clusters in ${\mathcal {C}}_{MSSC}$ and to different clusters in ${\mathcal {C}}_{true}$. The score ranges from 0 to 1, and a high value indicates a good similarity between the two clusters.

### Intrinsic measures

Available methods in Python:

- *Calinski-Harabasz Criterion (CHC)*^38^, also known as the Variance Ratio Criterion.
  The score is defined as the ratio of the between-the-cluster dispersion and the within-the-cluster dispersion. Good clustering has a large variance between the cluster and a small variance within the cluster, hence a large CHC.
- *Davies Bouldin Index (DBI)*^39^, which is defined as follows. Let $C_{r}, r\in [k]$, be the *r*-th cluster and $s_r$ the average Euclidean distance between the data points $p_i$ from cluster $C_r$ and the centroid of that cluster, denoted by $c^r$:
  8 $$\begin{aligned} s_r = \left( \frac{1}{|C_r|}\sum _{i\in C_r} \Vert p_i -c^r\Vert _2^2\right) ^{1/2},\forall r\in [k]. \end{aligned}$$
  The DBI score is defined as
  9 $$\begin{aligned} \textrm{DBI} = \frac{1}{k}\sum _{i=1}^k\max _{i\ne j \in [k]}\left( \frac{s_i+s_j}{\Vert c^i-c^j\Vert _2}\right) , \end{aligned}$$
  Therefore, DBI can only have non-negative values, and the smaller the value of DBI, the better the clustering.
- *Silhouette Evaluation Score* ($S_{score}$)^40^, defined as
  10 $$\begin{aligned} S_{score} = \frac{1}{n}\sum _{i=1}^n\frac{b_i-a_i}{\max \{a_i,b_i\}}, \end{aligned}$$
  where $a_i$ is the mean distance between the *i*-th data point and the other data points of the same cluster. Similarly, $b_i$ is the mean distance between the *i*-th data point and all other data points of the nearest cluster. The fraction $\frac{b_i-a_i}{\max \{a_i,b_i\}}$ thus measures how well the *i*-th data point is positioned in the cluster to which it is assigned. Thus, the value of $S_{score}$ is between $-1$ and 1, and the closer it is to 1, the more appropriate the clustering.

## Numerical results

### Datasets

As benchmark datasets, we used datasets collected by Tomas Barton and available in GitHub (https://github.com/deric/clustering-benchmark/tree/master/src/main/resources/datasets). These datasets consist of a subset of real data and a subset of artificial data. For all these datasets, the data providers have also provided the so-called ground truth clustering, i.e., the number of clusters and the classification into this number of clusters according to certain similarity criteria. We were not aware of these similarity criteria, probably they cannot be described explicitly, i.e. in the form of a mathematical distance.

From both datasets, we selected only those for which we were capable of solving (MSSC) for the values of $k\ge 2$ with $|k-k_{true}|\le 2$. Therefore, we exclude the instances where the number of data points or $k_{true}$ was too large (i.e., $n\cdot k_{true}>1000$). Nevertheless, we tried to solve also few instances with $n\cdot k_{true}>1000$ and the computing times were huge (more than a week on one computing node with 2x AMD EPYC 7402 24-core processors and 128 GB DDR4-3200 ram), but we managed to solve five such instances: dermatology, ecoli, glass, 3MC, lsun. 

The information about the selected datasets is summarized in Tables 2 and 3, where *n* is the number of data points, *m* is the number of variables (features), including the categorical variable containing the labels of the ground truth clustering in the data samples, and $k_{true}$ is the clustering number for the ground truth clustering.

All these datasets are formally numerical, which means that there is no categorical value in any dataset. However, a closer look reveals that some real datasets include also variables that are by their nature categorical, like heart-statlog and zoo, which contain half or even all variables that are categorical by their nature, respectively. We did not take this fact into account. The exact clustering results are included in the GitHub repository, see https://github.com/shudianzhao/GroundTruth_VS_OptimalCluster.

**Table 2:** Summarized information for real data.

Instances	Type	*n*	*m*	$k_{true}$	Measuring type
Dermatology	Real	358	35	6	Mixed
Ecoli	Real	336	8	8	Numerical
Glass	Real	214	10	7	Numerical
Haberman	Real	306	4	2	Numerical
Heart-statlog	Real	270	14	2	Mixed
Iono	Real	351	35	2	Numerical
Iris	Real	150	5	3	Numerical
Sonar	Real	208	61	2	Numerical
Tae	Real	151	6	3	Mixed
Thy	Real	215	6	3	Numerical
Wine	Real	178	14	3	Numerical
Zoo	Real	101	17	7	Categorical

**Table 3:** Summarized information for artificial data.

Instances	Type	*n*	*m*	$k_{true}$	Measuring type
3-spiral	Artificial	312	3	3	Numerical
3MC	Artificial	400	3	3	Numerical
Blobs	Artificial	300	3	3	Numerical
Flame	Artificial	240	3	3	Numerical
Gaussians1	Artificial	100	3	2	Numerical
Insect	Artificial	30	4	3	Numerical
Jain	Artificial	373	3	2	numerical
Lsun	Artificial	400	3	3	Numerical
Pathbased	Artificial	300	3	3	Numerical
Zelnik1	Artificial	299	3	3	numerical
Zelnik2	Artificial	303	3	3	numerical
Zelnik6	Artificial	238	3	3	numerical

### Results on real and artificial datasets

Table 4 and Table 5 contain the names of datasets (column instances) used in the computations, the value *k* for which (MSSC) was solved, the corresponding optimum values $d_{SOS\cdot }$ for (MSSC) (note that the rows highlighted in gray contain the feasible values of (MSSC), evaluated at ${\mathcal {C}}_{true}$). The rest of the columns contain the scores of the extrinsic and the intrinsic measures, described in “Quality measures” section, which were computed for the optimum clusterings ${\mathcal {C}}_{MSSC}$ and for ${\mathcal {C}}_{MSSC}$ (gray rows).

Column $d_{SOS\cdot }$ show that optimum values of (MSSC) decrease with *k*, as is expected - more groups enable smaller sum of squares of distances between the data points and the cluster centroids. For each dataset, we can observe that the corresponding value of $d_{SOS\cdot }$ in the gray rows, i.e., the values of (MSSC) evaluated at ${\mathcal {C}}_{MSSC}$, is usually much higher compared to the optimum value for $k=k_{true}$. This reveals that the ground truth clusterings ${\mathcal {C}}_{true}$ are often far from the optimum of (MSSC).

The best scores for each measure, evaluated on optimum clusterings, are highlighted in bold. All the measures we use have different preferences among these clustering results: different measures often achieve their best value for different *k*.

Moreover, the extrinsic measures rarely achieve the best score for $k=k_{true}$ (recall, $k_{true}$ is *k* in the gray rows) and the ground truth clusterings have scores for the intrinsic measures which significantly differ from the best scores, for both real and artificial data set.

**Table 4:** Clustering evaluations for real data.

instances	*k*	$d_{sos}$	extrinsic measurements						intrinsic measurements		
			AMI	ARS	*h*	*c*	NMI	FMS	CHC	DBI	$S_{score}$
Dermatology	6	71,575.5	1.00	1.00	1.00	1.00	1.00	1.00	20.68	5.90	$-$0.03
Dermatology	4	16,162.4	0.07	0.03	0.08	0.09	0.08	**0.25**	**558.07**	**0.89**	**0.35**
Dermatology	5	13,364.9	0.09	0.04	0.10	0.11	0.11	0.24	523.20	0.94	0.32
Dermatology	6	11,698.0	0.09	0.03	0.11	0.11	0.11	0.22	486.88	1.06	0.29
Dermatology	7	10,552.3	0.08	0.03	0.11	0.10	0.10	0.20	454.86	1.21	0.26
Dermatology	8	9,831.4	**0.15**	**0.06**	**0.20**	**0.16**	**0.18**	0.22	420.94	1.22	0.26
Ecoli	8	21.3	1.00	1.00	1.00	1.00	1.00	1.00	81.18	1.58	0.24
Ecoli	6	16.5	0.61	**0. 49**	0.65	**0.59**	0.62	0.62	**166.38**	1.26	**0.28**
Ecoli	7	15.0	0.61	0.53	0.68	0.58	0.62	**0.64**	157.93	1.23	0.27
Ecoli	8	13.8	0.60	0.43	0.71	0.55	0.62	0.56	150.13	1.22	0.25
Ecoli	9	13.0	0.63	0.44	0.76	0.57	0.65	0.58	142.48	1.16	0.26
Ecoli	10	12.3	**0.64**	0.44	**0.77**	0.57	**0.66**	0.58	135.71	**1.12**	0.27
Glass	6	911.2	1.00	1.00	1.00	1.00	1.00	1.00	19.70	3.74	$-$0.09
Glass	5	400.3	0.35	0.25	0.33	0.43	0.38	0.50	123.03	0.90	0.44
Glass	6	336.1	0.39	0.26	0.38	0.46	0.42	0.50	**124.62**	0.94	0.45
Glass	7	292.3	0.39	0.27	0.40	0.45	0.42	0.50	124.01	0.87	0.46
Glass	8	266.5	**0.42**	**0.28**	**0. 44**	**0.47**	**0.45**	**0.51**	118.85	**0.86**	**0. 46**
Haberman	2	53,123.2	1.00	1.00	1.00	1.00	1.00	1.00	8.36	4.73	0.06
Haberman	2	30,507.0	0.00	0.00	0.00	0.00	0.00	**0.55**	239.93	0.97	0.40
Haberman	3	21,164.6	**0.03**	**0.05**	**0.05**	**0.03**	**0.04**	0.53	239.22	**0.85**	**0. 43**
Haberman	4	15,366.5	0.02	0.03	0.04	0.02	0.02	0.44	**256.92**	0.85	0.37
Heart-statlog	2	934,653.4	1.00	1.00	1.00	1.00	1.00	1.00	11.14	4.16	0.05
Heart-statlog	2	549,315.0	0.02	**0.03**	0.02	**0.02**	0.02	**0.53**	**206.95**	**0.99**	**0.38**
Heart-statlog	3	431,736.0	0.01	0.01	0.02	0.01	0.01	0.43	167.52	1.16	0.28
Heart-statlog	4	351,403.0	**0.02**	0.02	**0.03**	0.02	**0.03**	0.42	156.97	1.08	0.28
Iono	2	3,086.2	1.00	1.00	1.00	1.00	1.00	1.00	17.75	4.09	0.15
Iono	2	2,419.4	0.13	0.18	0.14	0.13	0.13	**0.61**	**118.83**	**1.51**	0.30
Iono	3	2,193.3	0.19	0.22	0.24	0.16	0.19	0.59	83.28	1.80	0.29
iono	4	1,999.2	**0.23**	**0.24**	**0.34**	**0.18**	**0.24**	0.58	71.97	1.71	**0.31**
Iris	3	89.4	1.00	1.00	1.00	1.00	1.00	1.00	486.32	0.75	0.50
Iris	2	152.4	0.65	0.54	0.52	**0.88**	0.66	0.75	513.30	**0.40**	**0.68**
Iris	3	78.9	**0.76**	**0.73**	0.75	0.76	**0.76**	**0.82**	**560.40**	0.66	0.55
Iris	4	57.3	0.72	0.65	0.81	0.65	0.72	0.76	529.40	0.78	0.50
Iris	5	46.5	0.69	0.61	**0.82**	0.60	0.69	0.73	494.09	0.81	0.49
Sonar	2	351.6	1.00	1.00	1.00	1.00	1.00	1.00	6.00	5.69	0.03
Sonar	2	280.5	0.01	0.01	0.01	0.01	0.01	**0.50**	**59.70**	1.80	**0.20**
Sonar	3	235.0	0.00	0.00	0.00	0.00	0.00	0.41	55.29	**1.69**	0.19
Sonar	4	217.2	**0.02**	**0.02**	**0.03**	**0.02**	**0.02**	0.39	45.30	1.91	0.17
Tae	3	38,345.8	1.00	1.00	1.00	1.00	1.00	1.00	1.98	9.13	$-$0.02
Tae	2	21,803.3	0.00	0.00	0.01	0.01	0.01	**0.41**	**120.04**	1.05	**0.38**
Tae	3	16,775.4	0.01	0.01	0.03	0.03	0.03	0.36	99.67	1.10	0.34
Tae	4	13,221.3	0.02	0.01	0.04	0.04	0.04	0.31	96.91	1.10	0.34
Tae	5	10,537.7	**0.03**	**0.01**	**0.06**	**0.04**	**0.05**	0.28	99.87	**0.99**	0.36
Thy	3	38,859.2	1.00	1.00	1.00	1.00	1.00	1.00	68.80	1.24	0.38
Thy	2	41,608. 0	0.12	0.05	0.12	0.14	0.13	0.54	115.05	1.07	0.35
Thy	3	28,560.2	**0.49**	**0.58**	**0. 49**	**0.50**	**0.49**	**0.81**	131.84	0.92	**0. 46**
Thy	4	20,082.7	0.31	0.28	0.37	0.28	0.32	0.62	154.09	**0.82**	0.41
Thy	5	15,402.5	0.32	0.28	0.43	0.27	0.33	0.58	**165.92**	0.83	0.39
Wine	3	5,232,632.4	1.00	1.00	1.00	1.00	1.00	1.00	15.81	2.81	0.07
Wine	2	4,543,750.0	0.42	0.37	0.33	**0.59**	0.43	**0.66**	**19.49**	**2.18**	**0.11**
Wine	3	2,370,690.0	**0.42**	**0.37**	0.43	0.43	**0.43**	0.58	18.64	4. 30	0.04
Wine	4	1,331,900.0	0.38	0.30	0.43	0.36	0.39	0.52	11.96	7.74	$-$0.03
Wine	5	916,379.0	0.40	0.31	**0.50**	0.35	0.41	0.51	14.74	5.41	$-$0.06
Zoo	7	278.0	1.00	1.00	1.00	1.00	1.00	1.00	23.90	1.86	0.30
Zoo	5	158.6	0.67	0.58	0.66	0.74	0.70	0.68	**82.22**	**0.93**	0.43
Zoo	6	134.8	0.72	0.66	0.75	0.74	0.75	0.74	79.97	0.93	0.45
Zoo	7	119.7	0.75	0.71	0.81	0.75	0.78	0.77	76.22	1.02	0.44
Zoo	8	109.3	0.78	0.73	0.86	0.76	0.81	0.79	72.06	1.06	0.44
Zoo	9	100.1	**0.81**	**0.75**	**0.91**	**0.77**	**0.83**	**0.81**	69.13	0.99	**0. 45**

**Table 5:** Clustering evaluations for artificial data.

Instances	*k*	$d_{sos}$	Extrinsic measurements						Intrinsic measurements		
			AMI	ARS	*h*	*c*	NMI	FMS	CHC	DBI	$S_{score}$
3-spiral	3	30,109.4	1.00	1.00	1.00	1.00	1.00	1.00	5.80	5.88	0.00
3-spiral	2	19,577.3	**0.00**	0.00	0.00	0.00	0.00	0.40	184.66	1.17	0.35
3-spiral	3	12,286.9	$-$0.01	$-$0.01	0.00	0.00	0.00	0.33	238.31	0.88	**0.36**
3-spiral	4	9,200.4	$-$0.01	$-$0.01	0.00	0.00	0.00	0.28	245.93	**0.88**	0.35
3-spiral	5	7,352.1	0.00	**0.00**	**0.01**	**0.01**	**0.01**	**0.26**	**249.36**	0.89	0.35
3MC	3	2,056.3	1.00	1.00	1.00	1.00	1.00	1.00	628.05	0.71	0.50
3MC	2	3,204.8	0.45	0.33	0.37	0.57	0.45	0.61	665.38	0.72	**0.53**
3MC	3	1,929.4	**0.81**	**0.80**	**0.81**	0.80	**0.81**	**0.87**	682.43	0.70	0.50
3MC	4	1,221.7	0.73	0.61	0.83	0.65	0.73	0.73	793.14	0.71	0.49
3MC	5	786.8	0.76	0.64	0.94	**0.64**	0.76	0.75	**975.91**	**0.63**	0.53
Blobs	3	149.6	1.00	1.00	1.00	1.00	1.00	1.00	575.43	0.61	0.54
Blobs	2	337.0	0.58	0.50	0.47	0.77	0.58	0.72	346.80	0.79	0.48
Blobs	3	144.8	**0.86**	**0.90**	**0.86**	**0.86**	**0.86**	**0.94**	**599.50**	**0.59**	**0.56**
Blobs	4	123.0	0.76	0.76	0.85	0.69	0.76	0.84	486.26	0.84	0.46
Blobs	5	105.5	0.69	0.62	0.85	0.59	0.70	0.74	436.16	0.98	0.36
Flame	2	3,543.5	1.00	1.00	1.00	1.00	1.00	1.00	110.39	1.16	0.33
Flame	2	3,123.8	0.40	**0.45**	0.41	0.39	0.40	**0.74**	157.21	1.12	0.38
Flame	3	1,913.6	0.48	0.46	0.64	0.39	0.49	0.70	202.72	0.80	0.41
Flame	4	1,211.5	**0.54**	0.43	**0.83**	**0.40**	**0.54**	0.67	**258.16**	**0.70**	**0.44**
Gaussianl	2	0.6	1.00	1.00	1.00	1.00	1.00	1.00	2,000.66	0.19	0.86
Gaussianl	2	0.6	**1.00**	**1.00**	**1.00**	**1.00**	**1.00**	**1.00**	**2,000.66**	**0.19**	**0.86**
Gaussian1	3	0.5	0.80	0.75	1.00	0.67	0.80	0.87	1,352.79	0.69	0.63
Gaussian1	4	0.3	0.68	0.54	1.00	0.52	0.68	0.73	1,205.24	0.95	0.38
Insect	3	7,671.2	1.00	1.00	1.00	1.00	1.00	1.00	45.77	0.83	0.39
Insect	2	10,683.5	0.55	0.47	**0.46**	0.75	0.57	0.69	60.27	**0.58**	**0.55**
Iinsect	3	6,002.8	0.62	**0.62**	0.64	0.65	**0.64**	**0.74**	**62.25**	0.70	0.47
Insect	4	4,236.1	**0.57**	0.45	0.69	0.55	0.61	0.60	60.24	0.80	0.44
Insect	5	3,075.3	0.57	0.47	0.75	**0.53**	0.62	0.61	62.20	0.63	0.49
Jain	2	29,856.3	1.00	1.00	1.00	1.00	1.00	1.00	279.48	0.90	0.40
Jain	2	22,208.8	0.36	**0.32**	**0.40**	0.33	0.37	**0.70**	503.48	0.78	**0.50**
Jain	3	12,650.1	0.36	0.26	0.53	0.28	0.36	0.61	580.56	**0.70**	0.49
Jain	4	8,763.1	**0.41**	0.25	0.69	**0.29**	**0.41**	0.58	**611.76**	0.79	0.48
Llsun	3	449.4	1.00	1.00	1.00	1.00	1.00	1.00	384.44	0.71	0.48
Lsun	2	670.3	0.65	**0.67**	0.54	0.81	0.65	**0.82**	385.69	0.94	0.46
Lsun	3	381.6	0.54	0.44	0.54	0.54	0.54	0.64	487.95	0.76	0.50
Lsun	4	202.5	0.72	0.59	0.83	0.64	0.72	0.73	728.30	**0.56**	0.57
Lsun	5	143.9	**0.80**	0.65	**1.00**	**0.67**	**0.80**	0.77	**806.96**	0.58	**0.57**
Pathbased	3	22,689.2	1.00	1.00	1.00	1.00	1.00	1.00	51.91	2.53	0.26
Pathbased	2	13,638.3	0.49	0.40	**0.40**	0.63	0.49	0.65	**371.06**	0.75	0.52
Pathbased	3	8,957.9	0.54	**0.46**	0.51	0.58	**0.55**	**0.66**	359.10	**0.67**	**0.54**
Pathbased	4	7,230.3	0.46	0.38	0.50	0.43	0.47	0.58	319.18	0.87	0.44
Pathbased	5	5,901.9	**0.46**	0.41	0.55	**0.41**	0.47	0.58	308.88	0.94	0.38
Zelnik1	3	13.5	1.00	1.00	1.00	1.00	1.00	1.00	0.01	120.43	$-$0.01
Zelnik1	2	9.6	0.12	0.03	0.10	0.16	0.12	0.46	120.66	1.30	0.31
Zelnik1	3	6.9	0.16	0.05	0.16	0.16	0.16	0.40	142.07	1.06	0.33
Zelnik1	4	5.5	0.20	0.10	0.23	0.18	0.21	0.39	143.76	1.07	0.33
Zelnik1	5	4.1	**0.38**	**0.20**	**0.45**	**0.34**	**0.38**	**0.46**	**166.24**	**0.84**	**0.40**
Zelnik2	3	27.2	1.00	1.00	1.00	1.00	1.00	1.00	50.53	2.28	0.47
Zelnik2	2	18.9	0.50	0.42	**0.41**	0.65	0.50	0.67	279.02	0.70	0.57
Zelnik2	3	11.9	0.54	0.47	0.50	0.60	0.55	0.67	309.26	0.60	0.63
Zelnik2	4	8.7	0.55	0.49	0.55	0.56	0.56	0.68	317.89	**0.59**	0.65
Zelnik2	5	6.4	**0.61**	**0.57**	0.66	**0.57**	**0.61**	**0.71**	**350.92**	0.60	**0.68**
Zelnik6	3	6.0	1.00	1.00	1.00	1.00	1.00	1.00	191.44	1.56	0.57
Zelnik6	2	3.8	0.60	0.58	**0.49**	0.76	0.60	0.77	751.29	0.35	0.73
Zelnik6	3	2.5	0.64	0.65	0.58	0.71	0.64	0.79	631.33	**0.51**	0.74
Zelnik6	4	1.4	0.67	0.71	0.66	0.69	0.67	0.82	**824.47**	0.42	**0.76**
Zelnik6	5	1.1	**0.68**	**0.73**	0.70	**0.67**	**0.68**	**0.83**	806.81	0.47	0.74

We denote by $k_{opt}$ the value of *k* for which the given quality measure reaches its best value. We can observe that $k_{opt}$ is not necessarily equal to $k_{true}$. Table 6 summarizes the difference $|k_{true}-k_{opt}|$ across all real and artificial datasets, for all quality measures. We can see that ARS has $|k_{true}=k_{opt}|$ for 58.33% of the artificial datasets and FMS has $|k_{true}=k_{opt}|$ for 50.00% of the real datasets and for $|k_{true}=k_{opt}|$ for 58.33% of the artificial datasets. All other measures have $k_{true}\ne k_{opt}$ for more than 50% of the datasets.

**Table 6:** Distribution of the differences $|k_{true}-k_{opt}|$ across all measures values, separately for real and artificial data.

Measure	Real data			Artificial data		
	$|k_{true}- k_{opt}|$			$|k_{true}- k_{opt}|$		
	0	1	2	0	1	2
	(%)	(%)	(%)	(%)	(%)	(%)
AMI	25.00	8.33	66.67	25.00	16.67	58.33
ARS	25.00	16.67	58.33	58.33	8.33	33.33
*h*	16.67	25.00	58.33	16.67	0.00	83.33
*c*	8.33	8.33	83.33	33.33	33.33	33.33
NMI	8.33	8.33	66.67	41.67	0.00	58.33
FMS	50.00	25.00	25.00	58.33	8.33	33.33
CHC	41.67	16.67	41.67	25.00	16.67	58.33
DBI	25.00	41.67	33.33	33.33	41.67	25.00
$S_{score}$	25.00	33.33	41.67	41.67	25.00	33.33

### Visualizations

**Fig. 1:**
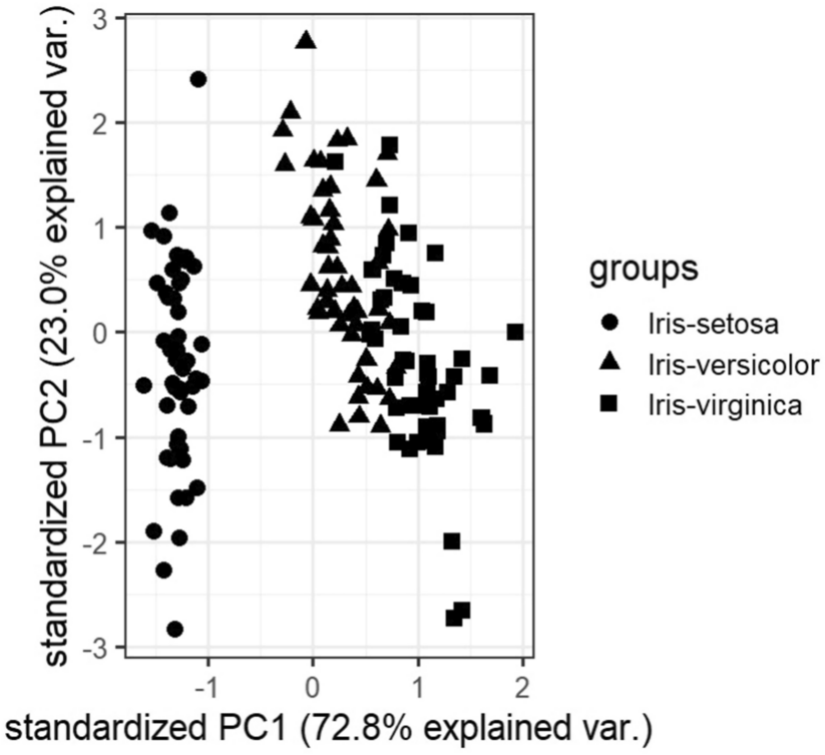
PCA visualization of ground truth clusters for the iris dataset.

**Fig. 2:**
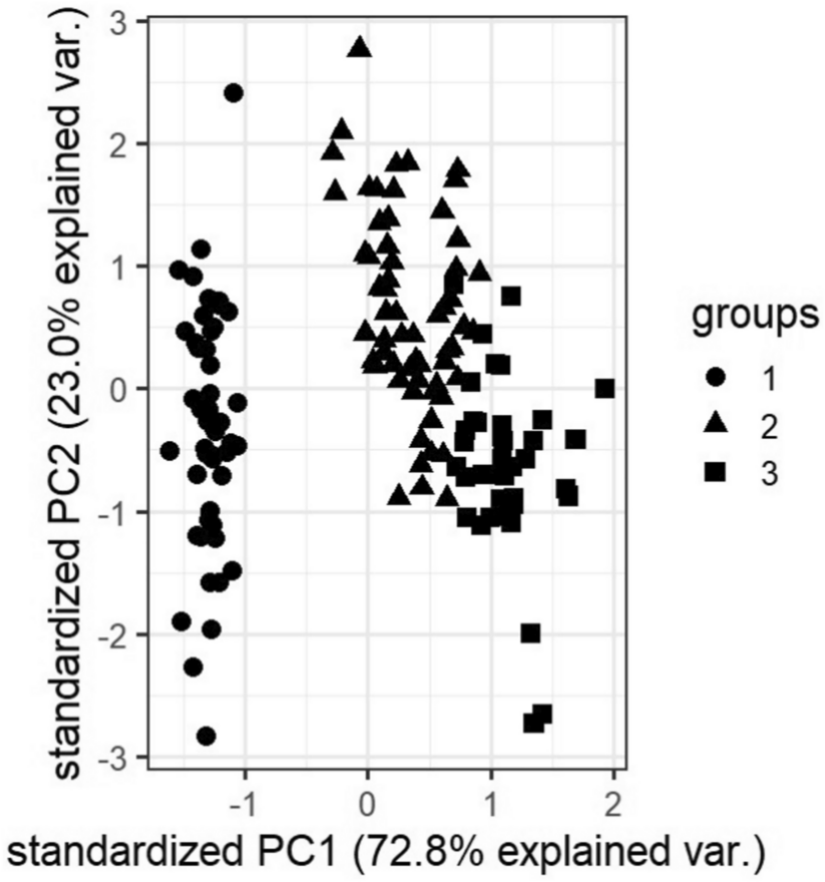
PCA visualization of optimum clusters for $k=k_{true}$ for the iris dataset.

**Fig. 3:**
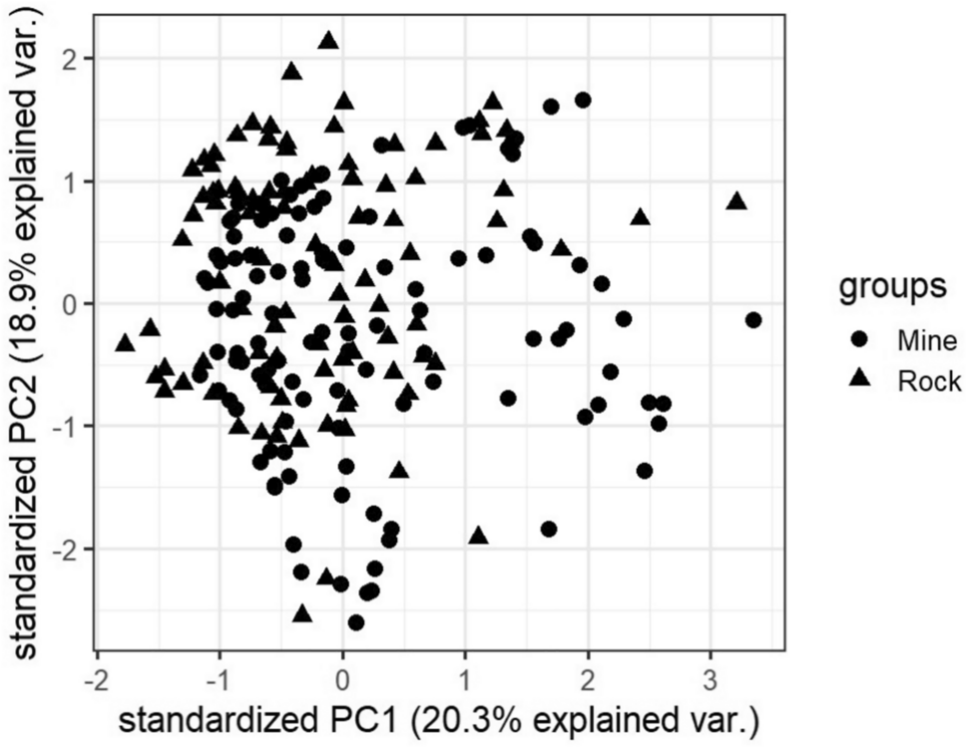
PCA visualization of ground truth clusters for the sonar dataset.

**Fig. 4:**
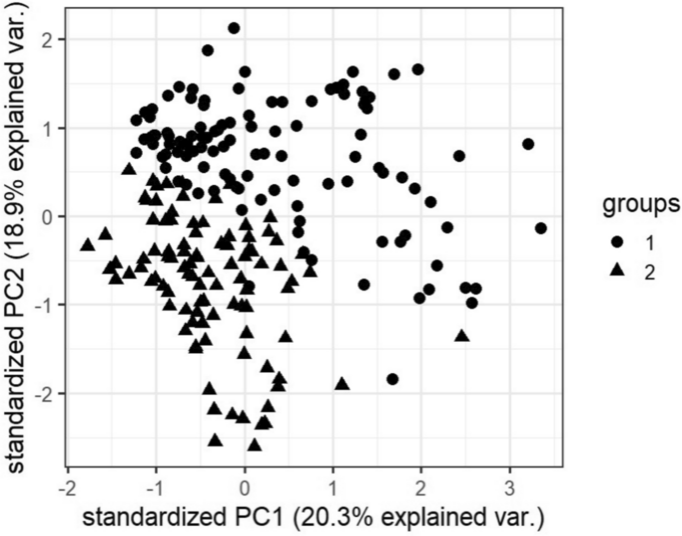
PCA visualization of optimum clusters for $k=k_{true}$ for the sonar dataset.

**Fig. 5:**
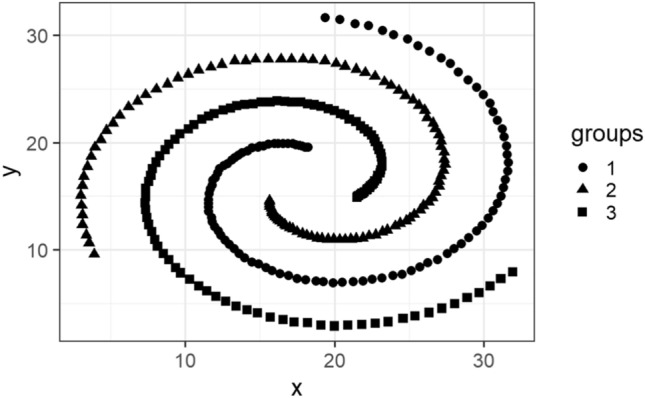
Visualization of ground truth clusters for 3-spiral dataset.

**Fig. 6:**
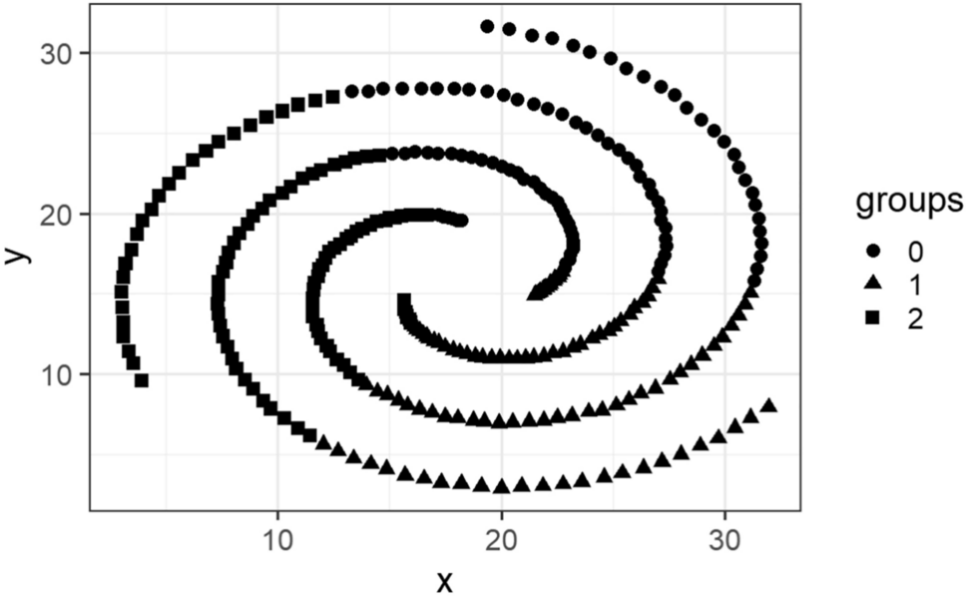
Visualization of optimum clusters for $k=k_{true}$ for 3-spiral dataset.

**Fig. 7:**
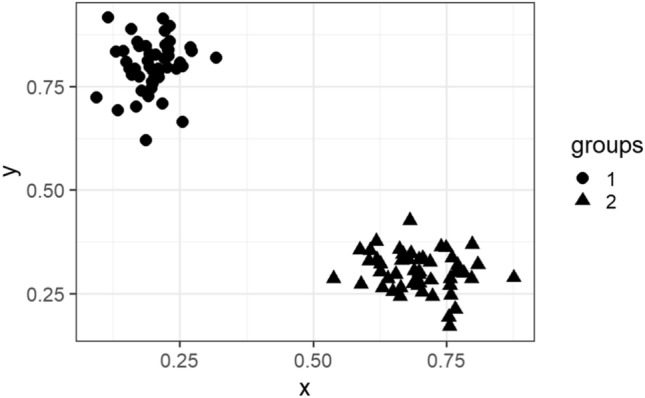
Visualization of ground truth clusters for gaussians1 dataset.

**Fig. 8:**
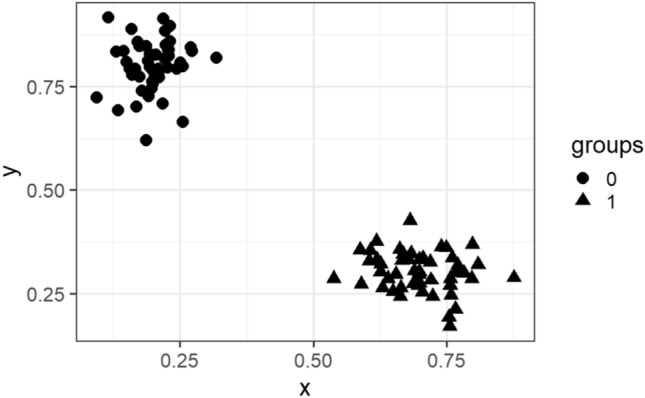
Visualization of optimum clusters for $k=k_{true}$ for gaussians1 dataset.

In this subsection, we visualize the ground truth and optimum clusterings for four datasets using principal component analysis (PCA): the iris and the sonar datasets from the real dataset group and the 3-spiral and the gaussians1 datasets from the artificial dataset group. These are shown in Figs. 1, 2, 3, 4, 5, 6, 7, 8. We have chosen these four because they represent extreme cases: For two of them (iris, gaussian1), the ground truth clusters have an ellipsoidal shape and are well separated in the two-dimensional space spanned by the two principal components, whereas for the other two data sets, the ground truth clusters are either highly overlapping (sonar) or have a non-ellipsoidal geometry (3-spiral).

For the iris and gaussian1 datasets, the ground truth and optimum clusterings are very similar, which we can realize by looking at the quality measures for $k=k_{true}$ in the corresponding tables. For the other two datasets, the quality measures are very poor, which means that the ground truth and the optimum clustering are very different. For the iris and sonar datasets, which have 4 and 60 dimensions, respectively, only the first two principal components are visualized, while the 3-spiral and gaussians1 datasets are two-dimensional, so the visualizations are completely correct. We can see that the ground truth clusters for the iris and the Gaussian1 are in the form of ellipsoids that are well separated, so the optimum and the ground clusterings are very similar. For the other two datasets, the ground truth clusters overlap or have the shape of spirals, and optimum clustering is completely different.

## Discussion

The numerical results from Tables 4 and 5 reveal several interesting facts. The main one is that the ground truth clustering usually does not match the optimum MSSC clustering. This has already been observed by other authors^16^, but our work shows it very clearly.

As first, even detecting $k_{true}$ is very challenging. We expected that the most popular quality measures for clustering from “Quality measures” section would have the best value for $k=k_{true}$. This would justify the usual approach where we detect $k_{true}$ by computing clusterings for different *k* by using some of the popular heuristic algorithms and then estimate $k_{true}$ to be the value for which some of the quality measures have the best value.

Table 6 shows that this is wrong. None of the quality measures can be used to detect $k_{true}$ for MSSC for the great majority of datasets. The FMS has the best value for $k=k_{true}$ for 50.0% of real datasets and for 58.3% of artificial datasets, while ARS has the best value for $k=k_{true}$ for 58.3% of artificial datasets. All the other measures achieve the best values for $k\ne k_{true}$ in more than 50 %. This means that we can not rely on these quality measures to detect $k_{true}$. Also the optimum value of (MSSC) can not be used to detect $k_{true}$ since this value is monotonically decreasing with *k* increasing, as is depicted in the 3rd columns of Tables 4 and 5. It is also interesting that the objective value of (MSSC), which is obtained on the ground truth clustering, is usually far from the optimum value for $k=k_{true}$, which means that the ground truth clustering is a feasible solution for (MSSC), which is in most cases far from the optimum.

We note that the intrinsic measures need special attention: all of them are related to Euclidean distance, but none of them measures exactly the same thing as we do in the objective function of (MSSC). They compare the within-the-group distances to between-the-group distances in different ways, whereas the objective function of (MSSC) only considers within-the-group distances. This is also a possible reason why these measures usually have the best value at different *k*, also different from $k_{true}$.

The only exceptions are the gaussians1 dataset, where the ground truth and the optimum clustering are the same, and the iris dataset, where the ground truth and the optimum clustering are similar but not the same. These clusterings are visualized in Figs. 1, 2 and 7, 8. These figures actually describe the numerical results very well. If the ground truth clusters have the expected geometry, i.e., the clusters have the form of convex sets, ellipsoids, which are well separated from each other, then such ground truth clustering is very similar to optimum clustering. Otherwise, if the clusters have geometrically different shapes, such as 3-spirals, or are overlapping, then the ground truth clustering is far from the optimum clustering, which always enforces the ellipsoidal geometry of the clusters since the Euclidean distance underlies this model.

Datasets ecoli, zoo, and thy also deserve some attention. For the first two datasets, all extrinsic measures are relatively high (higher compared to the other datasets, above 0.6), but the highest values are obtained for different values of *k*, also different from $k_{true}$. For the thy dataset, all extrinsic measures are highest for $k=k_{true}$, but these values are around 0.5 (for FMF it is 0.8). The intrinsic measures for these three datasets are somewhat convoluted, with their best values obtained for different *k*, with no apparent pattern. We also reviewed the geometry of the ground truth and the optimal clusters in the space spanned by the two principal components. The figure is not included, but it shows that the ground truth clusters overlap strongly in this two-dimensional space, but the geometry of the points allows for an ellipsoidal shape of clusters. It appears that in the original 8-dimensional space, the ground truth clusters are sufficiently separated from each other so that the optimal clusters are close to them. Moreover, the ground truth clusters are of very different sizes, 5 of them are large ($\ge 20$) and three of them are small ($\le 5$), and this is probably the reason why the extrinsic measures reach their optimum either for smallest *k* or for largest *k*.

In common clustering practice, we do not know ground truth clustering, but we still want to know how good is the clustering we obtained with a clustering algorithm. In such a case, we can rely on the intrinsic measures. Our results show that the choice of the intrinsic measure is crucial. Different measures tend to give very different answers about the quality of the clustering. Thus, before making a choice, we should understand very well what exactly (what kind of similarity) the chosen intrinsic measure measures, and make a choice on that basis.

Our work therefore confirms the conclusions of^16^ that the datasets with known ground truth, which are usually used as benchmark datasets for classification problems, can be used for benchmarking the clustering algorithms with careful attention, since the class labels are usually assigned based on the properties of each individual data point, probably including additional information not present in the dataset itself, while the clustering algorithms take into account the relationships (similarity) between the data.

## Conclusions

In this paper, we considered the mathematical programming formulation of the NP -hard minimum sum-of-squares clustering problem (MSSC) and solved it to optimality for a number of real and artificial datasets for which the ground truth clustering (the clustering created by the data provider) was available and which were of small size (the number of data points times the number of true clusters $k_{true}$ had to be approximately less than 1000). We solved these instances for the number of clusters *k* equal or close to the ground truth $k_{true}$ using the exact solver SOS-SDP^15^.

For each dataset and each *k* that we used we compared the optimum clustering (optimum solution of (MSSC)) with the ground truth clustering by using a number of extrinsic and intrinsic measures.

We showed that the ground truth clusterings are usually quite far from the optimum clustering, for all *k* close to $k_{true}$, which means that (i) they yield the value of the objective function of (MSSC), which is usually much worse (higher) compared to the optimum value of (MSSC), (ii) the values of intrinsic measures evaluated at the ground truth clustering are usually much worse than the values of the intrinsic measures evaluated at the optimum clustering, (iii) the values of the extrinsic measures which we used to measure the alignment between the ground truth and the optimum clustering showed that they differ a lot. However, if the ground truth clustering has a natural geometry, i.e., if the clusters look like ellipsoids that are well separated from each other, then the ground truth clustering and the optimum clustering are very similar.

We can derive the following main conclusions: (i) The ground truth clusterings were defined by data providers who often used similarity measures that were not based on Euclidean distance or on distances equivalent to this distance. It is likely that the similarity measure used was in fact not a distance (metric) according to the mathematical definition, see e.g.^43^. They may have even used additional information not included in the variables describing the data points. Therefore, there is most likely no mathematical distance at which the optimal value of (MSSC), where the objective function would be defined using this distance, would have ground truth clustering as the optimum solution. (ii) We should be very careful when comparing the clustering obtained by a particular clustering algorithm with the ground truth clustering. If such an algorithm measures the similarity between data points with a distance equal to the Euclidean distance, such as the famous *k*-means algorithm, while the ground truth clusters do not have an ellipsoidal geometry, then we cannot expect to obtain a solution that is close to the ground truth clustering. (iii) Determining the most appropriate number of clusters by considering where the values of the extrinsic or intrinsic measures have the best value can also be misleading. Very often these measures give contradictory answers: different measures suggest different *k*, which very often differ from $k_{true}$. The situation is somewhat better when the clusters have a natural ellipsoidal geometry. This confirms the great importance of choosing the similarity measure over the data points in data clustering: it should capture the geometry of the underlying data. Clustering should also be evaluated using quality measures that are aligned with the similarity measure used in the calculation.

In our future work, we aim to assess clusterings generated by algorithms that approximately solve (MSSC). These clusterings will be compared against those corresponding to the global optima of (MSSC). Furthermore, we will contrast both sets of clusterings with ground truth clusterings and those produced by well-established heuristic methods, such as *k*-means, agglomerative hierarchical clustering, and density-based clustering, among others. This comparative analysis may offer insights into the alignment between these different clusterings, as well as provide a deeper understanding of the performance trade-offs incurred when relying on standard heuristic approaches in lieu of (local) optimum clusterings.

## Data Availability

The data used for numerical experiments and all the exact clustering results are included in the GitHub repository (https://github.com/shudianzhao/GroundTruth_VS_OptimalCluster) and^25^.
